# Influence of Crosslinking Concentration on the Properties of Biodegradable Modified Cassava Starch-Based Films for Packaging Applications

**DOI:** 10.3390/polym16121647

**Published:** 2024-06-11

**Authors:** Sudarat Khadsai, Rapiphun Janmanee, Pornpat Sam-Ang, Yossawat Nuanchawee, Waleepan Rakitikul, Wilawan Mankhong, Wirot Likittrakulwong, Padarat Ninjiaranai

**Affiliations:** 1 Faculty of Science and Technology, Thepsatri Rajabhat University, Lopburi 15000, Thailand; sudarat.k@lawasri.tru.ac.th; 2 Department of Chemistry, Faculty of Science and Technology, Pibulsongkram Rajabhat University, Phitsanulok 65000, Thailand; rapiphun16@psru.ac.th (R.J.); pornpat.s@psru.ac.th (P.S.-A.); yossawat.n@psru.ac.th (Y.N.); 3 Program of Chemical Technology, Faculty of Science and Technology, Chiang Rai Rajabhat University, Chiang Rai 57100, Thailand; em_waleepan_r@crru.ac.th; 4 Department of Chemistry, Faculty of Science, Naresuan University, Phitsanulok 65000, Thailand; wilawanm@nu.ac.th; 5 Program of Animal Science, Faculty of Food and Agricultural Technology, Pibulsongkram Rajabhat University, Phitsanulok 65000, Thailand; wirotliki@psru.ac.th

**Keywords:** packaging, chitosan, citric acid, polysaccharide-based film, mechanical property

## Abstract

Chitosan/modified cassava starch/curcumin (CS/S/Cur) films with a crosslinker were developed via the solvent casting technique for the application of food packaging. The effects of citric acid (CA) as a natural crosslinker were assessed at different concentrations (0–10.0%, *w*/*w*, on a dry base on CS and S content). To measure the most favorable film, chemical structure and physical, mechanical, and thermal properties were investigated. Successful crosslinking between CS and S was seen clearly in the Fourier Transform Infrared (FTIR) spectra. The properties of the water resistance of the CS/S/Cur films crosslinked with CA were enhanced when compared to those without CA. Furthermore, it was found that the addition of CA crosslinking would improve the mechanical properties of composite films to some extent. It had been reported that the CA crosslinking level of 7.5 wt% of CS/S/Cur film demonstrated high performance in terms of physical properties. The tensile strength of the crosslinked film increased from 8 ± 1 MPa to 12 ± 1 MPa with the increasing content of CA, while water vapor permeability (WVP), swelling degree (SD), and water solubility (WS) decreased. An effective antioxidant scavenging activity of the CS/S/Cur film decreased with an increase in CA concentrations. This study provides an effective pathway for the development of active films based on polysaccharide-based film for food packaging applications.

## 1. Introduction

Packaging films based on biopolymers have attracted much attention for use in replacing synthetic petroleum-based plastic films which are harmful to both living organisms and the environment because they cannot biodegrade in the environment naturally after disposal [1]. Natural biodegradable polymers obtained from animals and plants, for example, lipids, proteins, and carbohydrates, have been utilized as materials sourced for a promising alternative to biodegradable films [2]. The main drawback of utilizing animal and plant proteins in the food industry is the high expense of large-scale industrial production. Lipids alone are not sufficient to generate cohesive films with good water vapor barrier characteristics. In contrast, the benefits of natural polysaccharides for use in plastic films consist of stable performance, good film-forming ability, abundant resources, and cost effectiveness [3].

Several polysaccharide-based biopolymers such as starch, alginate, agar, carrageenan, cellulose derivatives, and chitosan have been utilized in packaging films [4]. Starch is a natural polymer with repeating units of D-glucose that consist entirely of molecules of amylopectin and amylose [5]. Biopolymers are currently visually appealing materials for use in food packaging because of their excellent mechanical and film-forming qualities. However, the use of starch-based films for food packaging applications has been limited because of their relatively hydrophilic character, including high water sensitivity and barrier properties in poor moisture conditions [6]. The blending of bio-based polymers (chitosan, cellulose acetate, κ-carrageenan, etc.) and starch has been determined to achieve enhanced physical properties of composite materials [7].

Chitosan is a biopolymer derived from the deacetylation process of chitin [8]. It is a natural cationic, biodegradable, and biocompatible polymer. It is suitable for film formation due to its low cost and has excellent chemical resistance. The hydrophilic groups in its chemical structure decrease the water vapor barrier and mechanical properties [9]. Previous research has investigated the possibility of the development of chitosan-based edible film, as it has proven antimicrobial activity as well as good mechanical properties, gas selective permeability (O_2_ and CO_2_), and film-forming ability [10]. Films based on chitosan and starch have been prepared from different starch sources, such as rice [11], corn [12], and wheat and potato [6]. According to the research by Bonilla et al. [13], chitosan/wheat starch composite films tended to achieve a notable increase in thicker conditions and yielded glossier films and apparent viscosity with the particle surface of the FFD. Moreover, the composite film possessed slightly increased water vapor permeability and mechanical properties compared to films that contained starch only. This is especially true when the chitosan ratio was increased in the films. However, one of the main drawbacks of the barrier and mechanical properties of polysaccharide-based films was their sensitivity to water; swelling and water absorption caused these properties to disintegrate, which prevented the films from being used in food packaging [6].

Crosslinking techniques have been employed to develop polysaccharide-based film properties which are related to the sensitivity of films to water [14]. Biopolymer films generally have poor mechanical and barrier properties, which restricts their practical use. One of the major disadvantages is the high water sensitivity of biopolymer films. This factor causes them to dissolve when in contact with water or disfigures their barrier and mechanical capabilities due to water swelling and absorption [15]. One of the main problems with polysaccharide-based films is their high sensitivity to water. This can be assessed using several techniques, for example through monitoring contact angles, solubility, moisture content, water activity, and sorption, and with water vapor permeability measurement [16]. Therefore, crosslinking is an excellent method for improving the performance and utility of starch-based food contact films, notably in terms of water sensitivity, which restricts a large number of possible uses as materials which come in contact with food. Crosslinking decreases polymer mobility. In general, it improves the mechanical and barrier properties as well as water resistance, leading to a decrease in swelling and water solubility [15].

Many crosslinking agents have been applied, mostly for polysaccharides such as boric acid [17], ferulic acid [18], and glutaraldehyde [19]. Nevertheless, the three hydroxyl groups in the unit of glucose are mainly located on the starch molecule. The tendency of the starch film to absorb moisture is attributed to hydrophilic hydroxyl groups through hydrogen bonds on the amylopectin and amylose molecules. The high permeability of water vapor and poor mechanical strength performance are the results of the high sensitivity of the material to moisture and poor moisture resistance to water. The poor moisture resistance and relatively high susceptibility of starch film to water, resulting from their hydrophilic nature, limit their suitability for packing applications. Therefore, chemical crosslinking agents are required to alter the hydrophilic properties of polymeric materials. The hydroxyl groups of glucose can react with multifunctional groups of crosslinking agents to form covalent bonds between starches and improve the behavior of starch film [15].

Citrus fruits contain the organic polycarboxylic acid known as citric acid (CA). It can be used in various areas, for example as a compatibilizer, hydrolytic agent, crosslinking agent, and plasticizer [20]. Various research works exploring the utilization of CA as a crosslinker for polysaccharide-based films to improve various barriers and physical properties have been conducted. Moreover, curcumin (Cur) is a polyphenolic compound that is extracted from the *Curcuma longa* L. (turmeric) rhizomes and has been long used in anti-arthritic, antimicrobial, antioxidant, and anticancer fields, among others [21]. Curcumin is the major active ingredient and the most valuable in turmeric. As is well known, a material made from hydrophobic di-phenolic is non-toxic in high doses [22]. Hence, the addition of curcumin into biocompatible polymers to develop a functional composite of materials has gained significant attention. As mentioned above, the use of polysaccharide-based films for food packaging applications conveys a high water vapor permeability (WVP) due to the hydrophilic nature of polysaccharides. WVP is frequently regarded as a crucial component of food packing and needs to be used as minimally as is feasible [23]. It is anticipated that the addition of curcumin to the film formulation will cause the WVP to decrease, as curcumin is a hydrophobic compound.

In previous research, studies have explored the production of composite films by a combination of CA crosslinking with chitosan/starch and curcumin. This research has aimed to examine the impact of varying concentrations of CA crosslinking agents on the mechanical, thermal, and water resistance characteristics of composite films. The identification will provide knowledge on suitable performances of composite films to be used for potential application in food packaging.

## 2. Materials and Methods

### 2.1. Materials

**Figure 1:**
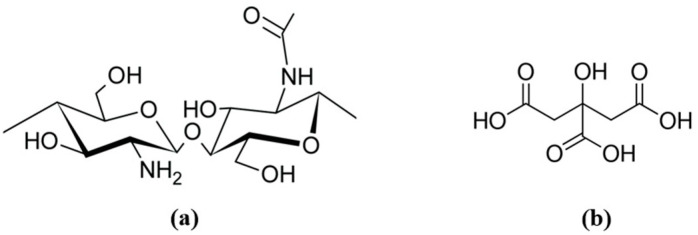
Chemical structures of (**a**) chitosan and (**b**) citric acid.

Modified cassava starch and curcumin were purchased from Krungthepchemi Co., Ltd. (Bangkok, Thailand). Chitosan was purchased from Tokyo Chemical Industry Co., Ltd. (Tokyo, Japan) (Figure 1a). DPPH was purchased from Sigma-Aldrich (Gillingham, UK). Citric acid (Figure 1b), glycerol, acetic acid, and ethanol were purchased from Merck (Merck, GA, USA). All the chemicals and reagents utilized were of analytical grade.

### 2.2. Sample Preparation

**Scheme 1:**
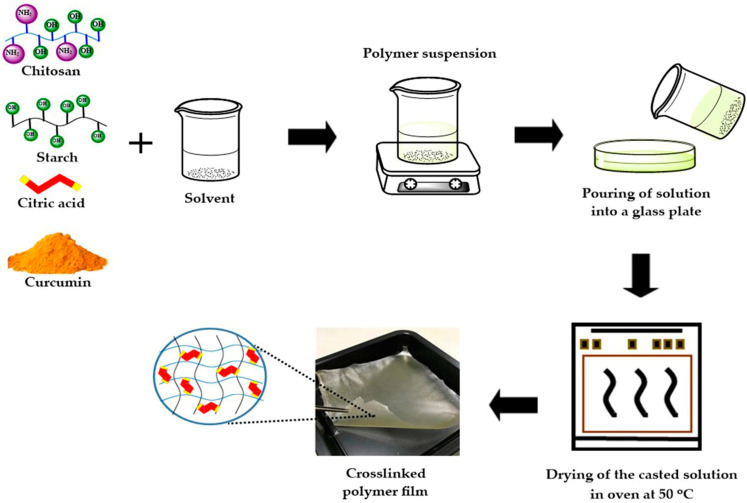
Synthesis scheme of the crosslinking reaction between chitosan, starch, citric acid, and curcumin.

Chitosan (CS, 2% *w*/*v*) was mixed with 1% *w*/*w* acetic acid solution by stirring at 90 °C for 5 h. A slurry of modified cassava starch (S, 2% *w*/*v*) was dissolved in deionized water at 70 °C for 30 min to complete the gelatinization process of the starch and then cooled down to room temperature. For the composite film preparation, 20% *w*/*w* (based on the total weight of CS and S) of glycerol was combined and magnetically stirred at 40 °C for 30 min. The blended chitosan/starch (CS/S) film was prepared at the ratio of 1:1 (*w*/*w*). As an active ingredient, 0.05 g of curcumin was combined with 10 mL of ethanol and added to the film-forming solution. Subsequently, 0%, 2.5%, 5.0%, 7.5%, and 10.0% (*w*/*w*) (based on the total weight of CS and S) of CA were added to the CS/S solution and further mixed for 30 min. Finally, 150 mL mixture solutions were cast on a glass plate and dried in an oven at 50 °C and 53% RH for 10 h (Scheme 1). The obtained sample films were assigned to CS/S/Cur, CS/S/Cur-2.5CA, CS/S/Cur-5.0CA, CS/S/Cur-7.5CA, and CS/S/Cur-10.0CA, respectively, depending on the CA used. The dried films were peeled easily from the glass plate and kept in a desiccator until further analysis. Code names and sample compositions are presented in Table 1.

### 2.3. Film Characterization

#### 2.3.1. Fourier-Transform Infrared (FTIR) Spectroscopy

A FTIR spectrophotometer (a Thermo Nicolet iS5, Norristown, PA, USA) was used to investigate the FTIR spectra of the composite films. At a spectral resolution of 4 cm^−1^ and 32 scans, the apparatus was set for detection in the 400–4000 cm^−1^ wavenumber range.

#### 2.3.2. Differential Scanning Calorimetry (DSC)

Samples of 5 mg were weighed and heated at a temperature range of 30 °C to 300 °C at the flow rate of 10 mL/min under a nitrogen atmosphere by a Mettler Toledo DSC 1 ((Columbus, OH, USA), software: STARe version 13)).

#### 2.3.3. Thermogravimetric Analysis (TGA)

TGA measurements were conducted using a Perkin Elmer Pyris TM 1 thermogravimetric analyzer (Perkin Elmer, New York, NY, USA). About 4–5 mg of the film sample was weighed into an aluminum pan and heated from 25 °C to 800 °C at a heating rate of 10 °C/min under nitrogen purge.

#### 2.3.4. Water Vapor Permeability (WVP)

The WVP of the composite films was employed gravimetrically using a WVP cup according to the ASTM E96-80 standard [24] method. Briefly, 15 mL of distilled water was placed in an aluminum cup, and the film sample (7 cm × 7 cm) was sealed on top of the cup and tightly closed. The film sample was conditioned at 50 ± 2% RH and 25 ± 2 °C. The weight loss of the cup was measured at intervals of 1 h for 24 h. The change in weight against time was carried out for regression analysis, and the line slope of the graph was received. The test was performed in triplicate [25].

#### 2.3.5. Moisture Content (MC), Swelling Degree (SD), and Water Solubility (WS)

Square pieces of film (4 cm × 4 cm) were cut and weighed ($M_{1}$), and then dried at a temperature of 100 °C for 4 h and reweighed ($M_{2}$). After that, the dried samples were subsequently immersed in a breaker containing 50 mL of distilled water at ambient temperature (25 ± 2 °C) for 1 day. Undissolved fragments of film were filtered out, and subsequently measured after the removal of excess water on the surface using blotting paper ($M_{3}$). Finally, the film samples were dried in an oven at a temperature of 105 ± 2 °C until the film mass became constant ($M_{4}$). The film values of MC (%), SD (%), and WS (%) were calculated as follows:

(1) $$\mathrm{MC}\;(\%)=\frac{M_{1}-M_{2}}{M_{1}}\times 100$$

(2) $$\mathrm{SD}\;(\%)=\frac{M_{3}-M_{2}}{M_{2}}\times 100$$

(3) $$\mathrm{WS}\;(\%)=\frac{M_{2}-M_{4}}{M_{2}}\times 100$$

#### 2.3.6. Film Thickness

The thickness was measured by a thickness micrometer (PEACOCK, dial gauge, OZAKI MFG. Co., Ltd., Tokyo, Japan), taking the average of 5 randomly selected positions. Triplicate films were conducted for each type of film.

#### 2.3.7. Mechanical Properties

The tensile strength (TS) and elongation at break (EB) of films were measured by the INSTRON^®^ CALIBRATION LAB model 5965 (software: Instron Bluehill v3.73.4823, Bluehill Universal, Norwood, MA, USA). Samples were cut into 100 mm × 10 mm strips. The testing conditions were a crossed speed of 50 mm/min with an initial grip length of 80 mm. The film measurements were operated with five replications, which were evaluated involving tensile strength (MPa) and elongation at break (%).

#### 2.3.8. Antioxidant Property

The antioxidant activity of film samples was determined using the DPPH (2,2-diphenyl-1-picrylhydrazyl) radical scavenging method [26]. Each film sample (about 25 mg) was dissolved in 5 mL of ethanol for 3 h, followed by centrifugation for 20 min at 1500× *g*. Then, 2 mL DPPH solution was mixed with 2 mL of diluted sample and incubated in the dark for 30 min at room temperature. After incubation, the absorbance value of film samples was measured using a UV–vis spectrophotometer at 517 nm, and the films’ antioxidant activity was calculated according to Equation (4), below [27,28]:

DPPH scavenging activity (%) = [(A_control_ − A_sample_)/A_control_] × 100 (4)

where A_control_ and A_sample_ are the absorbance values of the DPPH solution without and with a mixture of film extract solution.

#### 2.3.9. Statistical Analysis

All film formulations were replicated three times and analyzed by one-way analysis of variance (ANOVA) with the Statistical Package for the Social Sciences, SPSS Version 17 (SPSS, Armonk, NY, USA). The differences (*p* < 0.05) were calculated using Duncan’s test.

## 3. Results

### 3.1. Structural Characterization

#### 3.1.1. FTIR Spectra

**Figure 2:**
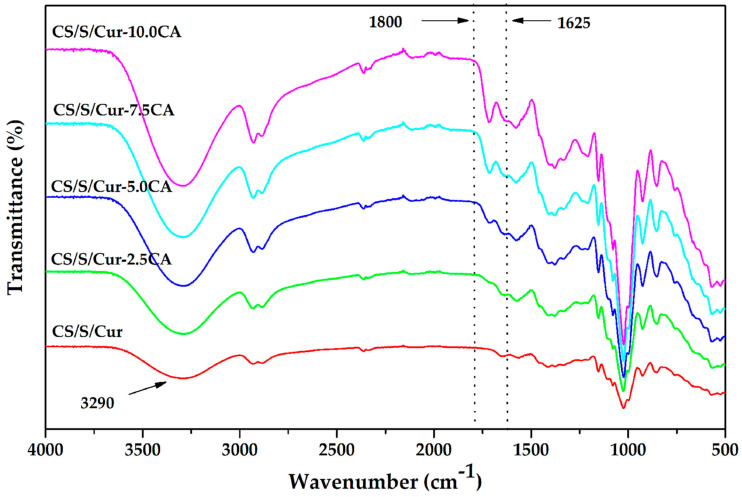
FTIR spectra of CS/S/Cur composite films crosslinked with and without CA.

FTIR was used to identify the molecular interactions and the functional chemical groups of the crosslinked biopolymer films. The FTIR spectra of the sample films without and with CA-incorporated films at the different concentrations are presented in Figure 2. The typical fingerprint region of carbohydrate components has been attributed to wave numbers up to 900 cm^−1^ and 1200 cm^−1^ in FTIR spectra. Moreover, adsorbed polymer was indicated by the peak at 1200 cm^−1^ [29].

A new characteristic peak was observed at a wavenumber of 1720 cm^−1^ after adding CA to the composite film. The CA spectra demonstrated the characteristic infrared absorption bands in the range of 1625–1800 cm^−1^. Those peak intensity measurements increased as the crosslinking concentration increased. This could be a result of the esterification between either chitosan or starch and CA, exhibiting chemical linkage formation among them [30]. Values of around 3200 cm^−1^ were associated with the C=O group and the -OH group of stretching vibration of the carboxylic acid. For the composite film without CA, the absorption peaks in the range of 3200–3400 cm^−1^ showed the stretching frequency in groups of -NH_2_ and -OH. The peak of the OH group observed around 3290 cm^−1^ in all films was due to the blending of chitosan and starch. Furthermore, CS/S/Cur films were dominated by a peak at the region around 3300 cm^−1^ caused by the OH group, which was wider and less intense for all films treated with CA. This is because there are OH groups that can easily vibrate as a result of the formation of hydrogen bonds [31].

#### 3.1.2. Thermal Analysis

**Figure 3:**
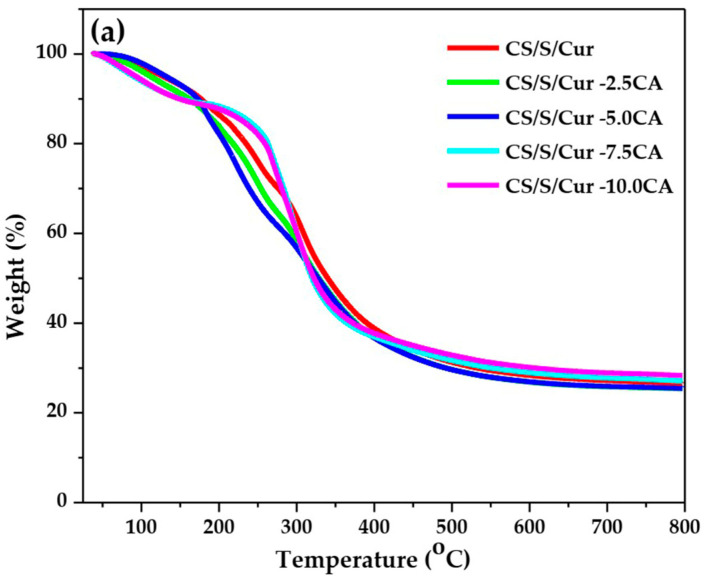

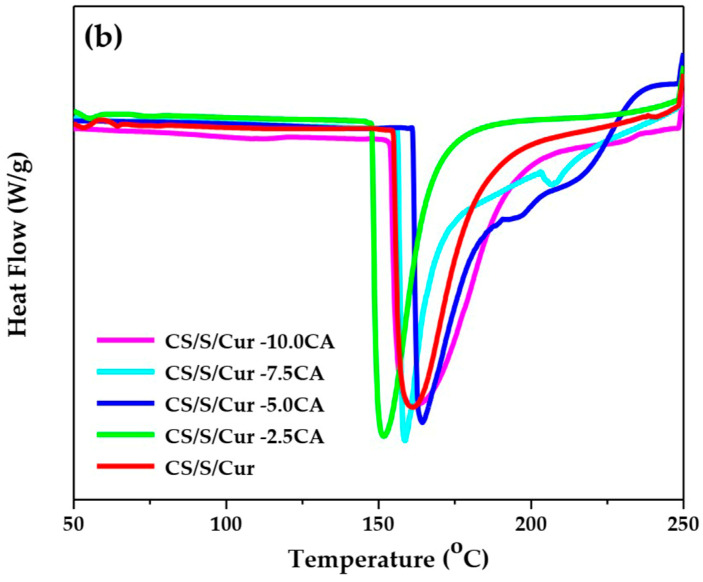
(**a**) TGA thermograms and (**b**) DSC thermograms of CS/S/Cur uncrosslinked film and CS/S/Cur crosslinked films with varying CA concentrations.

Thermogravimetry (TGA) is a thermal analysis technique in which the weight loss or gain of a film is measured as a function of time or temperature. Figure 3a displays the TGA curves from 25 °C to 600 °C of CS/S/Cur films crosslinked with and without CA. The main peak related to the decomposition of CA around 212 °C [32]. Weight loss was obtained around 200–400 °C, which was attributed to complex processes including the decomposition and depolymerization of the deacetylated and acetylated units of the polymer, and the dehydration of the saccharide rings [18,33]. The TGA pattern of the composite film without a CA crosslinker presented two stages of decomposition. In the first step, the first mass loss was observed according to the loss of water and other volatile material up to 105 °C. In the second step, the degradation of the composite film that appeared at around 200 °C was due to glycerol evaporation [34].

However, the number of degradation stages increased when CA was added. The CS/S/Cur with CA crosslinked showed three stages of degradation (I, between 50 °C and 150 °C; II, between 230 °C and 300 °C; and III, between 305 °C and 450 °C). The elimination of moisture was the initial stage in the degrading process for all films [35]. For CA crosslinked films, moisture loss was seen at higher temperatures; this suggests that the water molecules and the film form constituents increasing the film thermal stability due to hydrogen bonding [36]. The decomposed temperature of glycerol was 160–220 °C [37], while CA had two decomposition temperatures that were roughly between 170 °C and 260 °C [38]. The primary degradation processes of the films occurred in the last stages of degradation, which were between 340 °C and 380 °C. These degradation processes correlated with the decompositions of both CS and S. Depolymerization and CS degradation may be the cause of CS degradation and the breakdown of the units of D-glucosamine and N-acetyl-d-glucosamine [39]. The elimination of carbohydrate components in the backbone was also linked to this stage [40].

As shown in Figure 3b, the thermal profiles of polymer films with and without a crosslinker were determined by differential scanning calorimetry (DSC). The thermograms of the crosslinked film exhibited endothermic peaks in the range of 145–170 °C, while the thermogram of the uncrosslinked film displayed an endothermic curve which showed at about 162 °C. It was found that when CA was added to the composite films, the peak corresponded to water evaporation, and the maximum peak of evaporation tended to weaken and shifted from 50 °C to 120 °C during the second heating cycle. This is because of crosslinking interactions that dramatically hinder water evaporation [32].

### 3.2. Water Resistance Properties

#### 3.2.1. Moisture Content (MC), Swelling Degree (SD), and Water Solubility (WS)

**Figure 4:**
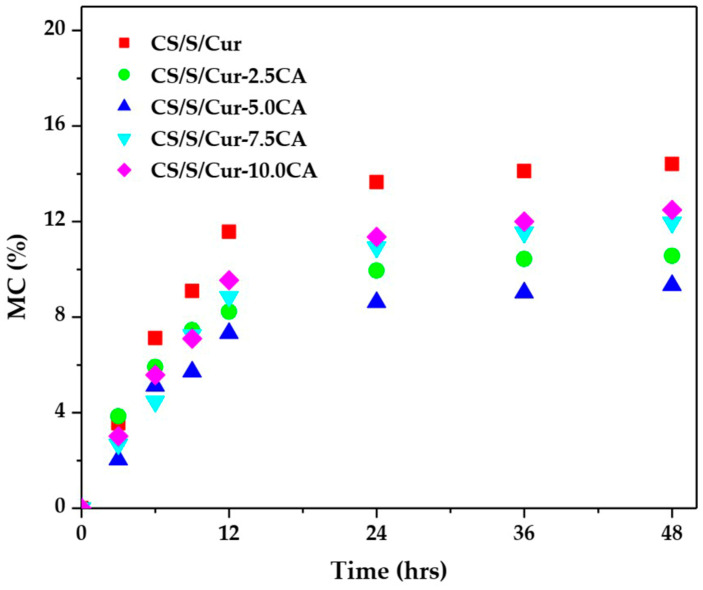
Moisture sorption curves of CS/S/Cur films without and with the CA crosslinker.

Moisture content is used to determine the moisture amount in the polymer films. As seen in Figure 4, the values of the moisture content significantly decreased from 15 ± 1% to 9 ± 1% in the CS/S/Cur films when increasing the CA concentrations, which was in agreement with the first step of TGA weight loss (Figure 3a). The rationale behind the reduction could be attributed to a decrease in the number of free hydrophilic groups present in the film as a result of crosslinking. In contrast, CS/S/Cur-5.0CA had the lowest moisture content when compared to CS/S/Cur-7.5CA and CS/S/Cur-10.0CA because the excess CA in the film matrix contained hydrophilic carboxyl groups. In the CA crosslinking mung bean starch film study, similar results were also reported by Yao et al. [41].

Water solubility (WS) is an important parameter for selecting biopolymers with potential applications. The main objective of biopolymer crosslinking is to reduce solubility and swelling with improved water resistance and applicability, and the results are shown in Table 2. The control film had a WS of 22 ± 1%, while the WS values of CS/S/Cur film with CA decreased from 21 ± 2% to 17 ± 2%. It was demonstrated that polymer films exhibited less solubility when the crosslinker was added. This may be due to the hydrophobic ester groups generated between polysaccharides and CA and the decreased hydrophilic availability of hydroxyl groups, resulting in enhanced insolubility and a condensed structure of the composite film with the crosslinker compared to the control film [42,43,44,45]. Alternatively, the WS value of CS/S/Cur crosslinked film increased once more with the excessive addition of 10 wt% CA. This rise is most likely due to the additional CA that could not crosslink with polymers, resulting in more hydroxyl groups and the attraction of water [46].

Moreover, Table 2 shows the swelling degree (SD) values of composite films with and without CA as a crosslinker. Crosslinking may result in decreased film solubility, according to the chemical interactions between the chitosan and starch molecules [44]. It has been reported that the SD of the CS/S/Cur films with the crosslinker was markedly lower than that of the control CS/S/Cur film (*p* < 0.05), resulting in a low free volume due to a strong interaction between CS and S. This could be explained by the generation of a dense structure with better resistance and decreased hydrogen bonding to water [45]. In particular, the SD of the crosslinked films considerably reduced from 76 ± 12% to 32 ± 1%.

#### 3.2.2. Water Vapor Permeability

**Figure 5:**
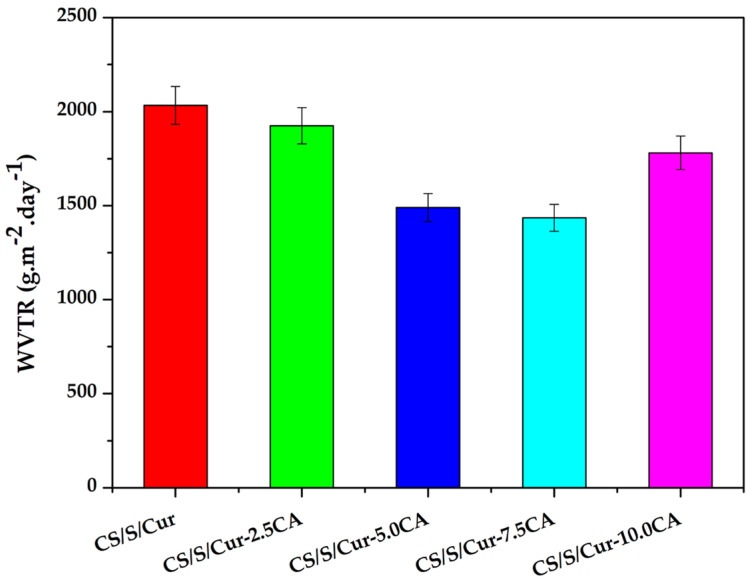
Water vapor transmission plot of CS/S/Cur uncrosslinked film and CS/S/Cur crosslinked films with different concentrations of CA crosslinker.

The water transmission rate (WVTR) of food packaging refers to the amount of moisture that can pass through a material. The packaging material acts as a barrier to moisture between the food and the surrounding environment, especially in the case of dry packaging. Figure 5 exhibits the WVTR of CS/S/Cur crosslinked films with and without CA. The WVTR of the CA crosslinked films ranged from 1435 ± 1 g·m^−2^·day^−1^ to 1925 ± 3 g·m^−2^·day^−1^, whereas the control film had a WVP of 2034 ± 1 g·m^−2^·day^−1^. It can be observed from the WVTR values that the control film was higher than the crosslinked film (*p* < 0.05) [30].

Similar results for CA crosslinked films causing a drop in WVTR were also reported by Seligra et al. [47]. The composite film would limit the swelling because the CA crosslink effect led to a strong interfacial interaction with a compact structure [3]. The addition of glycerol as the plasticizer into the film enhanced the flexibility of the starch film by decreasing the molecular force of polymer chains [41]. Usually, plasticizers containing hydrophilic compounds such as polyols (sorbitol and glycerol) are utilized in starch films and have been found to be particularly suitable for plasticized hydrophilic polymers [48]. Therefore, the hydrophilic hydroxyl groups of glycerol can improve the moisture sorption/desorption, leading to increased water vapor transmission [49].

Nevertheless, CA acts as a strong crosslinker that can reduce the availability of amine groups and free hydroxy in the film structure. Thus, denser network structures can further restrict the chitosan swelling and consequently decrease water vapor transmission through the film [45]. Mathew and Abraham [18] also studied a changing tendency in WVP values of the chitosan and starch-based composite films containing ferulic acid. Similar results were also obtained by Ghanbarzadeh et al. [46] in prepared starch-based films incorporating CA using casting method. The result is that the WVP values of a film decreased by 57% in 10% CA formulation compared with blends without a compatibilizer.

### 3.3. Physical and Mechanical Properties

#### 3.3.1. Thickness

The improvement of the physical properties of biopolymer composite films is a key objective for chemical crosslinking with CA. An increasing thickness in films contributes to the addition of CA in the composite film compared to the control film, and this change is more pronounced for the higher CA content (*p* < 0.05) in the sample films. Table 2 displays the thickness of composite films. The films had a thickness ranging from 0.12 mm to 0.14 mm. The film thicknesses are directly proportional to the formulation in the solid concentrations [44]. The incorporation of CA involved a high solid content in film-forming solutions, resulting in an increase in the film thickness. In addition, the composite film density increased with a more compact film structure when increasing in CA concentration, which was ascribed to the interaction of crosslinking, which could cause binding between polymers [41,50].

#### 3.3.2. Mechanical Properties

To ensure food safety and structural integrity during transportation, handling, and storage, food packaging material should have good mechanical characteristics. Evaluating the mechanical characteristics of films is essential since poor mechanical properties limit the commercial use of biopolymer-based films. Tensile strength (TS) and elongation at break (EB) are the parameters most often utilized to characterize the mechanical properties of films. TS is the maximum force that a material can withstand when stretched before breaking. The percentage of the film that has been extended to its starting duration is called EB [51].

**Figure 6:**
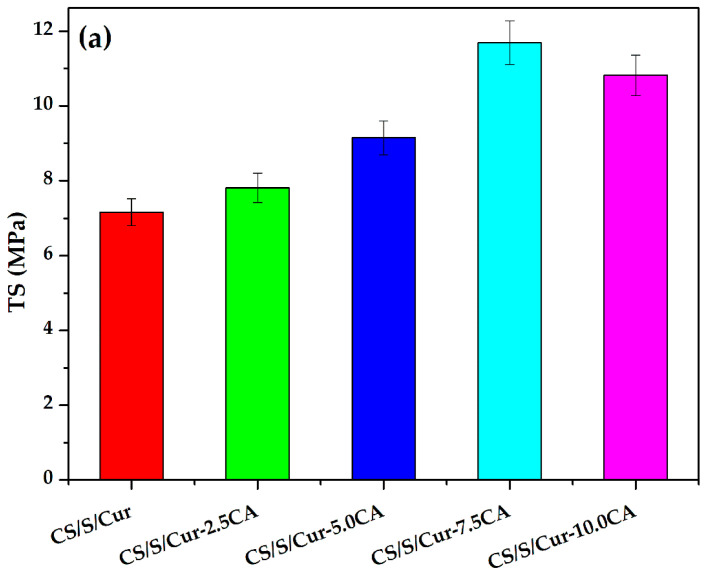

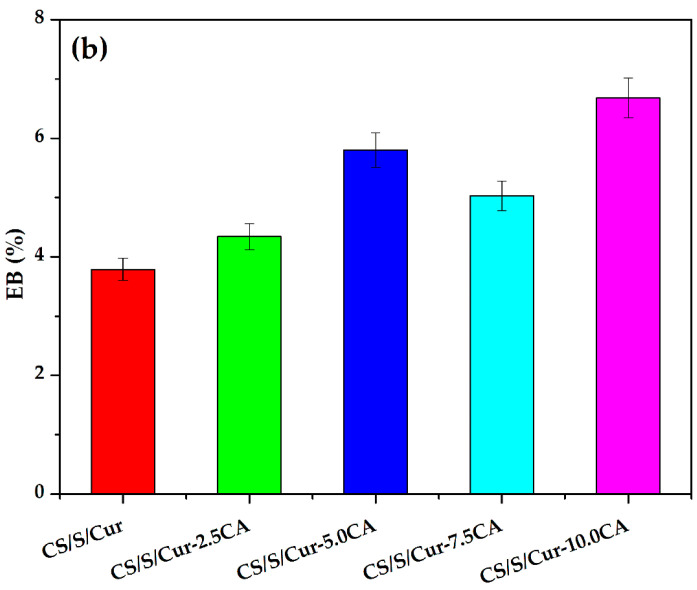
Mechanical properties of composite films with and without CA as a crosslinker: (**a**) TS and (**b**) EB.

The effects of the CA crosslinker on the tensile properties of CS/S/Cur films with and without CA including TS and EB were determined, and the data are presented in Figure 6a,b, respectively. The TS and EB of composite films with and without CA were 7 ± 1 to 12 ± 1 and 4 ± 1 to 7 ± 1, respectively. It can be noticed that the addition of CA led to a significant increase in rigidity because of its crosslinker function [52]. CA can serve as a plasticizer and crosslinker because its chemical composition consists of one hydroxyl and three carboxyl groups, providing a variety of functions [53].

In contrast, the CA in biopolymer films may act as both a plasticizer and a crosslinking agent. The additional portion of citric acid likely decreased the interactions between the macromolecules by acting as a plasticizer, as a result decreasing TS and increasing EB. Comparable results were found by Wang et al. [54]. Wang and team determined the effect of CA concentration with enhanced mechanical properties on polyvinyl alcohol/xylan films. The 10 wt% CA film had significantly higher TS than the control film. Nevertheless, a plasticizing effect caused the EB to increase and the TS to decrease when the CA concentrations were higher than 10 wt%.

In this study, TS increased with an increase in CA concentration while EB decreased with an increase in CA concentration. However, the addition of CA at a concentration of more than 7.5 wt% resulted in a decrease in TS and an increase in EB. It is well known that the tensile properties are affected by the opposite effects of the plasticizers and the crosslinking agents [55]. Similar results were obtained from other studies using CA-crosslinked biopolymers, which were explained by the fact that decreased TS was seen at 6 wt% CA because the mobility of starch molecules was limited by extensive crosslinking [45]. In another report, the addition of CA up to 10 wt% increased the tensile strength of starch-based films, whereas a concentration of CA exceeding 15 wt% resulted in a decrease in TS because of the plasticizing effect [46]. Figure 6b presents the relationship between the EB and CA crosslinker content of the CS/S/Cur composite films. In the case of CS/S/Cur-5.0CA, the elongation of break values follows a decreasing trend from 7 ± 1 to 4 ± 1% of CS/S/Cur-10.0CA to CS/S/Cur, respectively. The EB value of CS/S/Cur-7.5CA moderately decreased more than that of CS/S/Cur-5.0CA. These results may have been caused by the interactions between CA and chitosan or CA and starch, which could have increased the film’s rigidity and reduced its EB.

### 3.4. Antioxidant Activity

#### DPPH Radical Scavenging Activity

**Figure 7:**
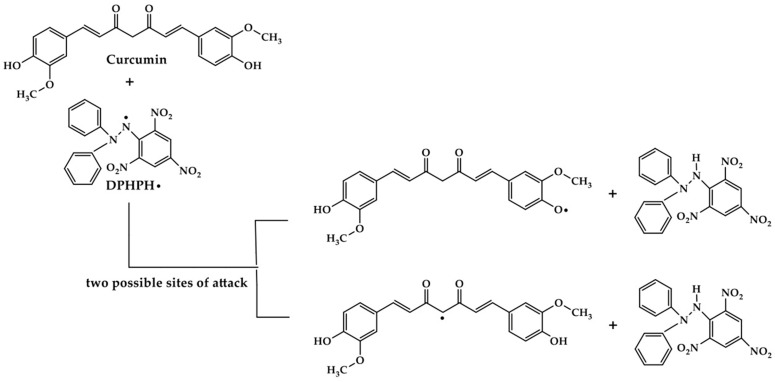
Chemical reaction between DPPH radicals and curcumin [59].

The antioxidant activity of composite films was evaluated using DPPH. Scavenging activity is a measure of the discoloration that results from the reaction between antioxidant radicals and DPPH reagents (Figure 7). The main components of the antioxidant activity of the composite film were identified as CS, CA, and Cur. Numerous studies have been conducted on the antioxidant activity of Cur. The combination of the active antioxidants curcumin and chitosan resulted in a considerable increase in the antioxidant capacity of the chitosan film when compared to the control sample since the active hydroxyl groups of chitosan can react with free radicals and show radical scavenging activity [56]. Curcumin is often incorporated into CS/S composite films, which was consistent with the curcumin release rate of the films. It is interesting to note that the antioxidant property originated from the methylene group of β-diketone moiety and the phenolic hydroxyl group in the structure of curcumin [27,57].

In this study, CA has the potential to be an antioxidant. When CA is added to CS films, the antioxidant activity can be increased. However, contrary to expectations, the increase in CA content in films in this study did not enhance the antioxidant activity of CS films. In other words, a rise in CA content resulted in a decrease in antioxidant activity (Table 3). This result was caused by the fact that when the concentration of CA increased, there were more hydrogen bonds between CS and CA. The radical scavenging activity diminishes with a decrease in the availability of free amino and hydroxyl groups from chitosan [55]. Furthermore, curcumin active sides might have reacted with the carboxyl group with CA crosslinking, leading to a reduction in the number of active sites, which are desirable for the effective antioxidant activity. The efficiency of the release of curcumin was characteristically inversely related to the crosslinking degree. A lower crosslinking density will give a higher release rate, in which the actual network structure will be formed and the crosslink process will take place [58]. Yildiz Ilhan et al. [27] discovered a similar result. It was observed that the antioxidant activity of faba bean/curcumin/CS films was reduced by CA crosslinking. The decrease in the number of active sides necessary for antioxidant activity was elucidated by the interaction between the carboxyl group of CA and the active sites of Cur, as well as the hydroxyl and amino groups of CS, during the crosslinking process.

## 4. Conclusions

CS/S/Cur films incorporating a citric acid crosslinker and glycerol plasticizer were successfully fabricated by the solution casting method in a simple and cost-effective process. The mechanical, water vapor barrier, and antioxidant properties of the CS/S/Cur films were affected by the incorporation of citric acid. The CS/S/Cur with citric acid had improved water resistance due to the factor of crosslinking. This resulted in lower values of moisture content, water solubility, and the water vapor barrier. The TS of the film increased, while EB decreased with an increase in citric acid concentration. Specifically, the results indicated that 7.5 wt% citric acid of CS/S/Cur film exhibited the optimal physiochemical and barrier properties. Thus, it is expected that these films can be utilized as a promising food packaging application.

## Figures and Tables

**Table 1:** Compositions of CS/S blending without and with CA as a crosslinker and curcumin.

Film Types	Compositions				
	Chitosan (CS) (g)	Modified Cassava Starch (S) (g)	Glycerol (%*w*/*v*)	Citric Acid (%*w*/*w*) *	Curcumin (mg/mL)
CS/S/Cur	2	2	20	-	50
CS/S/Cur-2.5CA	2	2	20	2.5	50
CS/S/Cur-5.0CA	2	2	20	5.0	50
CS/S/Cur-7.5CA	2	2	20	7.5	50
CS/S/Cur-10.0CA	2	2	20	10.0	50

* Based on the total weight of CS and S.

**Table 2:** Physicochemical of composite films with and without CA as a crosslinker.

Film Types	Physicochemical Properties		
	Thickness (mm)	Swelling Degree (%)	Water Solubility (%)
CS/S/Cur	0.12	148 ± 6	22 ± 1
CS/S/Cur-2.5CA	0.13	76 ± 12	21 ± 1
CS/S/Cur-5.0CA	0.14	50 ± 7	19 ± 2
CS/S/Cur-7.5CA	0.14	40 ± 5	17 ± 2
CS/S/Cur-10.0CA	0.14	32 ± 1	21 ± 2

**Table 3:** Antioxidant activity of films.

Film Types	DPPH (% Scavenging Activity)
CS/S/Cur	31 ± 0
CS/S/Cur-2.5CA	49 ± 1
CS/S/Cur-5.0CA	27 ± 1
CS/S/Cur-7.5CA	21 ± 1
CS/S/Cur-10.0CA	17 ± 0

## Data Availability

Data are contained within the article.
